# Ethnopharmacological Perspective for Treatment of Epilepsy: An Updated Review

**DOI:** 10.1155/2024/8052659

**Published:** 2024-11-20

**Authors:** Sunishtha Kalra, Saurabh Bhatia, Ahmed Al Harrasi, Syam Mohan, Himanshu Sachdeva, Divya Sharma, Vikas Budhwar, Manjusha Choudhary, Rohit Malik

**Affiliations:** ^1^Department of Pharmacology, Institute of Pharmaceutical Sciences, Kurukshetra University, Kurukshetra, Haryana 136119, India; ^2^Department of Pharmaceutical Sciences, Maharshi Dayanand University, Rohtak, Haryana 124001, India; ^3^Natural and Medical Sciences Research Centre, University of Nizwa, P.O. Box 33, PC 616, Birkat Al Mauz, Oman; ^4^School of Health Sciences, University of Petroleum and Energy Studies, Dehradun, Uttarakhand 248007, India; ^5^Substance Abuse and Toxicology Research Centre, Jazan University, Jazan 82912, Saudi Arabia; ^6^Center for Transdisciplinary Research, Department of Pharmacology, Saveetha Dental College, Saveetha Institute of Medical and Technical Science, Saveetha University, Chennai 602105, India; ^7^Department of Pharmacognosy and Phytochemistry, School of Pharmaceutical Sciences, Delhi Pharmaceutical Sciences and Research University, New Delhi 110017, India; ^8^Department of Pharmacology, ICFAI School of Pharmaceutical Sciences, The ICFAI University, Jaipur, Rajasthan, India; ^9^Department of Pharmacology, SRM Modi Nagar College of Pharmacy, SRMIST, Delhi-NCR Campus, Ghaziabad, India

**Keywords:** antiepileptic drugs, epilepsy, Ethnopharmacology, GABA, glutamate, traditional plants

## Abstract

Plants have been used as healing agents since humanity began. This review presents the plant profiles inhabiting the world regarding their traditional usage by various tribes/ethnic groups for the treatment of epilepsy. The bibliographic investigation was carried out by analyzing standard reference textbooks, Science Direct, Google Scholar, Scopus, Medline, Web of Science, and PubMed databases. Search terms and keywords used for the search were epilepsy, medicinal plants for epilepsy, herbal remedies used in the treatment of epilepsy, and traditional antiepilepsy medication. This review article was prepared by including the biological names of plants/their parts/extracts/compounds/doses/models/results. Further, experimentally explored 15 potential medicinal plants have also been explored in detail. The present review was prepared by including 114 plants from 3 books, 83 research, and 59 review articles. This review indicates that the list of medicinal plants presented in this review might be useful to researchers for preliminary screening of potential antiepileptic agents.

## 1. Introduction

Epilepsy is the most common neurological disorder, affecting up to 1% of the world population [1], (approx. 65 million people globally). Children are more commonly diagnosed than adults, with a frequency of nearly eight per 1000 children below the age of seven years [2]. It is the second most complex central nervous system disorder after stroke and is mainly effective in low- to middle-income countries [3] (WHO). According to the CDC, 1.2% of the US population has been affected by active epilepsy [4]. However, in India, approximately 10 million people are suffering from epilepsy, among them 3 million people have drug-resistant epilepsy and 1 million people require surgery [5]. The direct expenditure on epilepsy is estimated to cost $28 billion per year. In 2014, the yearly cost of epilepsy per individual was $15,414, according to a retrospective examination of US claims data; this comprised outpatient, inpatient, and treatment expenditures [6].

Epilepsy is mainly characterized by the loss of consciousness, jerking, and abnormal neuronal activity with or without convulsion due to exaggerated expression of neurons in the brain [7, 8]. Some serious disorders like stroke, genetic abnormalities, Hodgkin's disease, congenital defects, and brain tumors/infections are also linked with epilepsy [7, 8]. The risk of mood disorders and cognitive impairment has been rising because people who have epilepsy avoid day-to-day physical activities or exercises due to the fear of seizures [9]. It has been reported that about 24% of the epilepsy patients are dealing with mental health issues [7].

Although the exact etiology of epilepsy is not known, some genetic factors, sudden environmental change, weak immune system, brain injury, brain infection, and brain stroke could be the main causes of seizures. Seizures are triggered by a variety of direct and indirect events including fever, illness, certain medication use/withdrawal, alcohol, and hyponatremia [10]. Furthermore, seizures are of two types: partial seizures and generalized seizures. Partial seizures occur when seizure activity is widespread in a large part of one hemisphere or only a small area of a lobe and generalized seizures brain in both (left and right) hemispheres are affected [11]. Due to disturbance in normal membrane conductance, an imbalance occurs between inhibitory and excitability activity which produces generalized or partial seizures.

## 2. Pathophysiology and Drug Targets of Epilepsy

**Figure 1:**
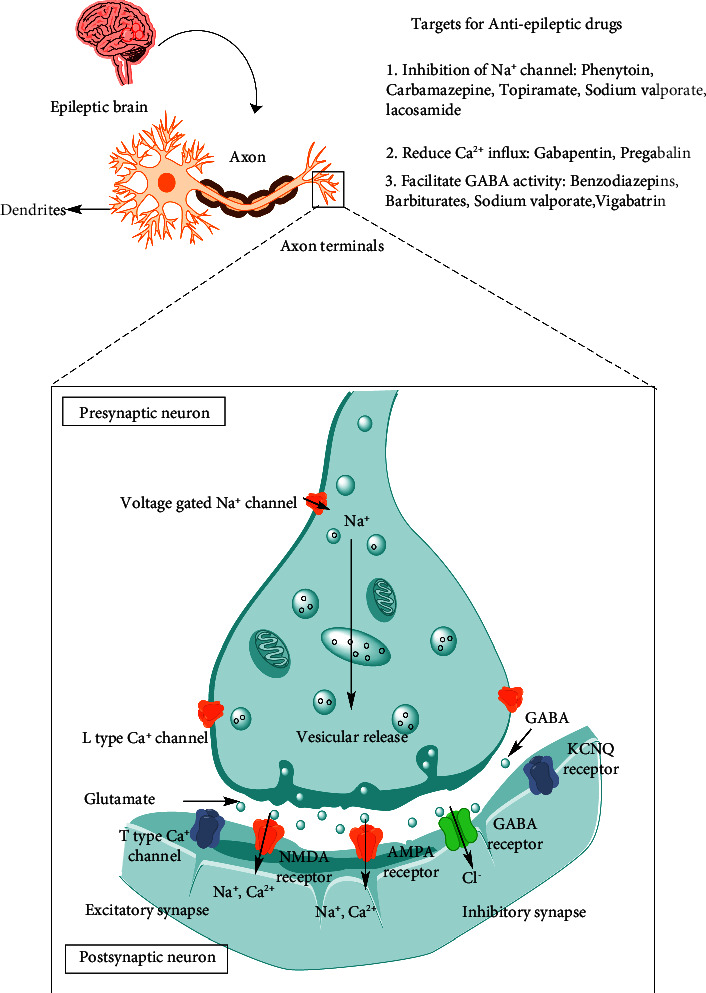
Pathophysiology of epilepsy and major targets for antiepileptic drugs.

Seizures can occur when there is distortion in the brain or when there is an imbalance between the excitation and inhibitions in the brain. This imbalance results in the alteration of brain functioning, from genes to signaling pathways and neuronal circuits. The other major cause is genetics. It leads to epilepsy from the circuit level to the receptor level. During normal brain development synaptic function (excitatory) develops before synaptic function (inhibitory), indulged in enhancing excitation and seizure generation GABA can also produce excitation than inhibition in the early period of life [12]. The pathophysiology of epilepsy is illustrated in Figure 1. Various synthetic drugs are also available in the market for the treatment of epilepsy and aim to suppress all types of seizures. Drugs like valproic acid, Lamotrigine, Carbamazepine, Oxcarbazepine, and Ethosuximide reduce epilepsy inpatients through various mechanisms, i.e., by increasing the concentration of GABA in the brain, by inactivation of the sodium channel in the brain, by decreasing post-tetanic potentiation, and by modulating T-type calcium gate. Regrettably, these marketed drugs fail to control seizures in patients and have shown various side effects that range from the severity of CNS impairment to death [13]. Also, they produce some unpleasant side effects and unpredictable pharmacological actions; therefore, it is necessary to search for newer drugs with fewer or no side effects [14]. So, newly there is more attention going towards herbal medicine in both western and developing countries. It is now becoming extremely apparent that besides these synthetic drugs, herbal or plant-based products meet the therapeutic demands of the population having mental health problems [15].

Synthetic antiepileptic drugs act mainly by three mechanisms: (i) decreasing electrical excitability of cell membrane; particularly by blocking the voltage-dependent sodium channels; (ii) increasing GABA-mediated synaptic inhibition; and (iii) inhibiting T-type calcium channels [16, 17].

## 3. Antiepileptic Screening Models

### 3.1. *In Vivo* Models

#### 3.1.1. Maximal Electroshock Seizure (MES) Model

The MES model is considered a gold standard for identifying potential therapeutic agents against generalized tonic-clonic seizures, which are the most common and severe form of seizures in humans [18]. In this model, seizures are induced in rodents (typically mice or rats) by delivering a brief, high-intensity electrical stimulus (50–60 Hz, 0.2–3 s) via transcorneal or auricular electrodes [19]. The electrical stimulus causes synchronized neuronal firing and generates a characteristic seizure pattern, consisting of an initial tonic phase of hindlimb extension, followed by a clonic phase of jerking movements [18].

Compounds with antiepileptic activity are evaluated based on their ability to prevent or delay the tonic hindlimb extension phase, considered the most critical component of the seizure response. The effectiveness of a compound is typically quantified by determining the median effective dose (ED50) required to prevent the tonic hindlimb extension in 50% of the animals tested. Additionally, the therapeutic index (ratio of the median toxic dose to the median effective dose) is often calculated to assess the safety margin of the compound [18].

The MES model is particularly valuable for identifying compounds that modulate voltage-gated sodium channels, which play a critical role in the initiation and propagation of seizures [18]. However, it is important to note that this model may not be effective in detecting compounds that act on specific neurotransmitter systems or mechanisms unrelated to sodium channel modulation [18].

#### 3.1.2. Pentylenetetrazole (PTZ) Seizure Model

The PTZ seizure model is widely used to evaluate the efficacy of compounds against myoclonic and generalized clonic seizures, which are associated with absence and generalized tonic-clonic seizures, respectively [20]. PTZ is a GABA receptor antagonist that induces seizures by inhibiting GABA-mediated inhibitory neurotransmission in the brain [18]. In this model, PTZ is typically administered systemically (subcutaneously or intraperitoneally) to rodents. The seizure response induced by PTZ is characterized by distinct phases, including initial myoclonic jerks, followed by clonic seizures, and eventually generalized tonic-clonic seizures [18]. Compounds with antiepileptic activity are evaluated based on their ability to delay or prevent the onset of these seizure phases or to increase the latency to various seizure stages.

The PTZ model is particularly useful for identifying compounds that modulate GABA-mediated inhibitory neurotransmission or other mechanisms related to absence or generalized tonic-clonic seizures [18]. However, it may not accurately reflect the pathophysiology of other types of seizures, such as partial or focal seizures [20].

#### 3.1.3. Kindling Model

The kindling model is a valuable tool for studying the process of epileptogenesis, which is the development and progression of epilepsy over time. In this model, repeated subconvulsive electrical or chemical stimuli are applied to specific brain regions (e.g., amygdala and hippocampus) of rodents. These stimuli initially cause brief, focal seizures, but with repeated stimulation, the seizures gradually become more severe and generalized, eventually leading to spontaneous recurrent seizures [18]. The kindling model is particularly useful for evaluating the efficacy of antiepileptic compounds in preventing or delaying the development of kindling, i.e., the progression of epileptogenesis. Compounds can be administered during the kindling process, and their effects on the severity, frequency, and duration of seizures can be assessed. Additionally, the model can be used to evaluate the ability of compounds to suppress spontaneous recurrent seizures in fully kindled animals, mimicking the treatment of established epilepsy [18].

The kindling model is a valuable tool for studying the long-term effects of antiepileptic compounds and for investigating the mechanisms underlying epileptogenesis and chronic epilepsy. However, it is important to note that the kindling process may not accurately reflect the pathophysiology of all forms of epilepsy, and the results may need to be interpreted cautiously in the context of specific epilepsy types [18].

### 3.2. *In Vitro* Models

#### 3.2.1. Hippocampal Slice Model

The hippocampal slice model is a widely used *in vitro* model for studying epileptiform activity and evaluating the antiepileptic potential of compounds. In this model, acute brain slices (typically 300–400 *μ*m thick) are prepared from the hippocampus or other brain regions of rodents or other species [21]. These slices are maintained in oxygenated artificial cerebrospinal fluid and kept viable for several hours. Epileptiform activity can be induced in these slices through various methods, such as the application of convulsants (e.g., bicuculline, a GABA receptor antagonist; 4-aminopyridine, a potassium channel blocker) or electrical stimulation [21].

The effects of potential antiepileptic compounds on the induced epileptiform discharges can be evaluated using electrophysiological recordings, typically through the use of extracellular or intracellular electrodes. Compounds that suppress or modulate the induced epileptiform activity are considered to have antiepileptic potential [21].

The hippocampal slice model is particularly valuable for studying the cellular and molecular mechanisms underlying epileptiform activity and for evaluating the effects of compounds on specific neuronal populations or circuits. However, it is important to note that the model lacks the complex interactions and modulation present in intact brain circuits, and the results may need to be interpreted cautiously in the context of the whole organism [21].

#### 3.2.2. Primary Neuronal Cultures

Primary neuronal cultures are another valuable *in vitro* model for studying epileptiform activity and evaluating the antiepileptic potential of compounds. These cultures are typically derived from dissociated neurons obtained from specific brain regions (e.g., hippocampus and cortex) of embryonic or neonatal rodents [22]. The dissociated neurons are plated on culture dishes or multielectrode arrays and allowed to develop and form functional neuronal networks over several weeks. Epileptic form activity can be induced in these cultures through various methods, such as the application of convulsants (e.g., bicuculline and 4-aminopyridine) or electrical stimulation [22].

The effects of potential antiepileptic compounds on the induced epileptiform discharges can be evaluated using electrophysiological recordings, typically using multielectrode arrays or patch-clamp techniques. Compounds that suppress or modulate the induced epileptiform activity are considered to have antiepileptic potential [22]. Primary neuronal cultures offer several advantages, including the ability to study epileptiform activity in defined neuronal populations and the ability to manipulate the culture conditions (e.g., growth factors, gene expression) to investigate specific mechanisms. However, like other *in vitro* models, the lack of complex brain circuitry and modulation may limit the translation of findings to the whole organism.

#### 3.2.3. Heterologous Expression Systems

In this model, specific ion channels or receptors known to be involved in epilepsy pathogenesis are heterologously expressed (i.e., expressed in a non-native cell type) in cell lines or expression systems, such as human embryonic kidney (HEK293) cells, *Xenopus laevis* oocytes, or other suitable hosts [23].

The cells or expression systems are genetically engineered to produce the target ion channel or receptor protein, which is then expressed on the cell membrane or incorporated into the appropriate cellular compartments. This allows for the precise study of the functional properties of these targets in a controlled and isolated environment, without the interference of other cellular components or signaling pathways present in native neuronal cells. The effects of potential antiepileptic compounds on the functional properties of these targets can be evaluated using various techniques, such as electrophysiological recordings (e.g., patch-clamp and two-electrode voltage-clamp), fluorescence-based assays, or biochemical assays. Compounds that modulate the activity of these targets in a manner expected to be antiepileptic (e.g., inhibiting excitatory ion channels, enhancing inhibitory ion channels, or receptors) are considered for further investigation [23].

Heterologous expression systems offer several advantages, including the ability to study the specific effects of compounds on isolated targets, the ability to introduce mutations or modifications to the target proteins, and the ability to perform high-throughput screening of compound libraries. However, it is important to note that these systems lack the complex cellular environment and interactions present in native neuronal cells, which may influence the functional properties and modulation of the targets [23]. Therefore, while heterologous expression systems provide valuable insights into the potential antiepileptic mechanisms of compounds, the findings from these systems often need to be complemented and validated using more physiologically relevant models, such as primary neuronal cultures or *in vivo* models, to better understand the potential therapeutic effects and translate the findings to the clinical setting.

It is worth noting that while each of these models has its strengths and limitations, a comprehensive evaluation of potential antiepileptic compounds often involves the use of multiple complementary models to gain a more complete understanding of the compound's mechanisms of action, efficacy, and potential therapeutic applications. The choice of models depends on the specific research questions, the stage of drug development, and the resources available [18, 23].

## 4. Methodology

The present study was conducted by investigating globally accepted databases such as books, recent research, review articles, and online databases, i.e., PubMed, Google Scholar, and Science Direct were searched. The information collected from 87 research and 60 review articles was reviewed and compiled. The biological corrected names of plants along with their families were verified from authentic sources and databases. Various medicinal plants that have antiepileptic activity are mentioned in Table 1. We have also explained in detail about 15 plant species for their proven use in the antiepileptic activity. The data of the present review was scrutinized by considering: (1) plants native to India, China, America, and Africa. (2) The plants used in epilepsy treatment in the Indian Ayurveda Medicine System. (3) The plants with proven antiepileptic activity, doses, model used mechanism of action and toxicity data. The study belonged to 62 families, among them Asteraceae, Fabaceae, Poaceae, and Apocynaceae are the main families. According to our study, most of the research work was carried out mainly in developing countries, but a significant contribution was also found to be seen in developed countries. It has also been observed that leaves and roots are maximally used for controlling the nervous system. Leaves (44%), roots (47%), bark (13%), fruits (11%), flowers (8%), seeds (11%), etc. have been used for the treatment of epilepsy Eight models have been used for the evaluation of antiepileptic activity and it was observed from our review that 4models were found to be most effective. Further, we included the literature published in the English language only, and *in vitro* studies, cell line studies, and chemical constituents are not included in this review.

## 5. Plants Have an Anticonvulsant Effect With the Mechanism of Action

### 5.1. *Ricinus communis* (RC) Linn

RC (Euphorbiaceae) commonly known as the castor oil plant is indigenous to India. Phytochemically, it contains steroids, saponins, alkaloids, flavonoids, and glycosides [93]. RC has been investigated for various pharmacological activities such as abortifacient, antiasthmatic, analgesic, antidiabetic [94], hepatoprotective, and anticonvulsant. Ethanol extract of RC leaves at doses of 200 and 400 mg/kg showed anticonvulsant activity when evaluated in MES and PTZ-induced seizure models in rats through modulation of GABAergic and glutamatergic neurotransmission, which are crucial for maintaining neural excitability and preventing seizures. It enhances GABA activity, an inhibitory neurotransmitter, thereby reducing neuronal excitability. Concurrently, it inhibits glutamate receptors, particularly NMDA receptors, which play a key role in excitatory neurotransmission. Additionally, RC affects voltage-gated Na^+^ and Ca^2+^ channels, reducing the influx of these ions and stabilizing neuronal membranes, further contributing to its anticonvulsant properties [91].

### 5.2. *Achyranthes aspera* (AA) Linn

AA (Amaranthaceae) annual or perennial herb found throughout the world, native to India is known as latjeera in Hindi and is traditionally used in asthma, cough, edema, dropsy, and piles [95]. Saponin A identified as D-glucuronic acid and saponins B identified as a β-D-galactopyranosyl ester of D-glucuronic acid were isolated from AA. Some other constituents were also isolated like oleanolic acid, amino acids, and hentriacontane. Methanol extract of plant roots at doses of 2.5, 5, and 10 mg/kg exhibited anticonvulsant activity in mice against MES, PTZ, and PIC-induced convulsions. The methanol extract of AA roots enhances the action of GABA, an inhibitory neurotransmitter, which reduces neuronal excitability and prevents convulsions. This effect is likely mediated through the upregulation of GABA receptors or the inhibition of GABA transaminase, thereby increasing GABA availability in the synaptic cleft. Additionally, AA might inhibit excitatory neurotransmission by blocking NMDA and AMPA receptors, further stabilizing neural activity [26].

### 5.3. *Punica granatum* (PG) Linn

PG (Punicaceae) is known as Pomegranate. It is a fruit-bearing shrub that has been investigated for various pharmacological activities like antidiabetic, antiobesity, antimicrobial, antidiarrheal, antibacterial, and neuroprotective [96]. Anticonvulsant activity of various extracts of the plant was investigated in MES and PTZ-induced seizure models in mice at doses of 150, 300, and 600 mg/kg, p.o. and indicates a reduction in the seizure's duration and a significant increase in the time of seizure onset in both the models. The saponins, flavonoids, triterpenes, and alkaloids in the seeds enhance GABAergic neurotransmission, increasing inhibitory effects on neurons and reducing excitability. These compounds may also inhibit glutamate receptors, decreasing excitatory neurotransmission. Furthermore, the antioxidant properties of these constituents help mitigate oxidative stress, protecting neuronal cells from damage and stabilizing neural function, which collectively contribute to the reduction in seizure duration and delay in seizure onset [35].

### 5.4. *Cardiospermum halicacabum* (CH) Linn

CH (Sapindaceae) is an annual or perennial climber widely distributed in India, tropical and subtropical Africa, commonly known as Kanphuti [97]. It is used in various countries in the treatment of rheumatism, stiffness of limbs, and snakebite whereas the root alone has been used for curing diseases related to the nervous system, and the seeds are used as a tonic for fever and as a diaphoretic. The alcohol root extract of CH at doses of 30, 100, and 300 mg/kg, revealed anticonvulsant activity in mice against MES, PTZ, PIC, STR, and ISO models. The alcohol root extract enhances GABAergic activity, increasing inhibitory signaling in the brain, which helps stabilize neuronal excitability and prevent seizures. This may involve upregulation of GABA receptors or inhibition of GABA degradation, thus increasing synaptic GABA levels. Additionally, the extract may modulate ion channels such as Na+ and Ca^2^+ channels, further contributing to its anticonvulsant properties by stabilizing neuronal membranes and reducing excitatory neurotransmission [47].

### 5.5. *Gmelina arborea* (GA) Roxb

GA (Verbenaceae) is known as Sewan in Hindi, native to the Philippines and Malaysia. It is widely distributed in various countries like China, Bangladesh, Asian countries, Myanmar, Thailand, Vietnam, Cambodia, and Indonesia. It contains various phytoconstituents like flavonoids, saponins, terpenoids, and cardiac glycosides are present in a major amount [98]. The anticonvulsant activity of methanol stem extract at 250 and 500 mg/kg doses was investigated against PTZ and STR models in mice using diazepam as a standard drug. GA exhibits anticonvulsant effects primarily through modulating GABAergic and glutamatergic neurotransmission. The methanol stem extract enhances GABAergic activity, increasing inhibitory signaling to reduce neuronal excitability. It likely involves upregulation of GABA receptors or inhibition of GABA transaminase, increasing GABA levels in the synaptic cleft. Additionally, GA may inhibit glutamate receptors, particularly NMDA and AMPA receptors, reducing excitatory neurotransmission. The glycine inhibitory properties further contribute by enhancing inhibitory synaptic transmission, stabilizing neural activity, and preventing seizures [99].

### 5.6. *Centella asiatica* (CA)

CA (Apiaceae) is a popular medicinal herb, commonly known as Brahmi, used in the management of the central nervous system, skin, and gastrointestinal disorders. Major chemical constituents of CA are triterpenes, Asiatic acid, and madecassic acid and their derived triterpene ester glycosides, asiaticoside, and madecassoside [100]. The anticonvulsant activity of the aqueous extract of CA was evaluated in induced seizures in albino mice at doses of 100 and 300 mg/kg. Extract at both doses suppressed the clonic seizures in mice but at a higher dose extract showed complete suppression of seizures and comparable anticonvulsant action to sodium valproate. CA exhibits anticonvulsant effects through multiple biochemical pathways. The extract likely enhances GABAergic neurotransmission by increasing GABA levels or receptor sensitivity, leading to greater inhibitory signaling and reduced neuronal excitability. Additionally, CA may modulate the cholinergic system, which influences cognitive functions and neuronal stability. The extract also affects ion channels, including Na^+^, K^+^, Mg^2+^, and Ca^2+^ channels, stabilizing neuronal membranes and reducing the likelihood of seizure propagation by regulating ion flow and maintaining cellular homeostasis [101].

### 5.7. *Acorus calamus* (AC) Linn

AC (Araceae) is a perennial herb, commonly known as sweet flag, native to central Asia and India. Phytochemically, it contains alpha-asarone, beta-asarone, and eugenol. Ethanol rhizome extract of AC at doses of 250 and 500 mg/kg, showed anticonvulsant activity in Swiss albino mice for MES and PTZ-induced seizure model. In the MES seizure model significantly decreased hind limb extension and tonic flexion of forelimbs were noted at both doses but in PTZ no significant activity was found [24]. AC demonstrates anticonvulsant activity primarily through the modulation of NMDA receptors. The extract, rich in *α*-asarone and *β*-asarone, particularly *α*-asarone, blocks NMDA receptors, which are critical for excitatory neurotransmission. By inhibiting these receptors, AC reduces excessive neuronal excitation and prevents the spread of seizures. This blockade likely decreases calcium influx through the NMDA receptor channels, stabilizing neuronal membranes and reducing the excitability that leads to convulsions, as evidenced by the significant reduction in hind limb extension and tonic flexion in the MES seizure model [102].

### 5.8. *Gossypium herbaceum* (GH)

GH (Malvaceae) is widely distributed in India, commonly known by the name of the cotton plant. Phytochemically, it contains steroids, alkaloids, carbohydrates, glycosides, tannins, and proteins [103]. Aqueous leaves extract GH at doses of 10, 30, and 100 mg/kg, p.o. showed protection in mice against MES, PTZ, and ISO-induced convulsions model. In the MES model, the extract exhibited dose-dependent antiepileptic activity and a more potent effect than diazepam. Also, in the PTZ model, both doses of the extract showed dose-dependent antiepileptic activity and were found to be more potent than standard drugs. Whereas in the ISO model, all three doses of the extract showed a dose-dependent delay in the onset of convulsions and were found to be less potent than diazepam. The aqueous leaf extract likely increases GABA levels or receptor sensitivity, enhancing inhibitory signaling and thereby reducing neuronal excitability. This modulation of GABAergic pathways stabilizes neural activity, leading to a dose-dependent reduction in seizure duration and delay in seizure onset in MES, PTZ, and ISO-induced convulsion models [79].

### 5.9. *Nigella sativa* (NS) Linn

NS (Ranunculaceae) is an annual plant commonly known as black cumin, used as herbal medicine for the treatment of various diseases like asthma, diarrhea, diabetes, and ulcers. Seeds of NS contain fixed oils, proteins, alkaloids, saponin, and essential oil. Thymoquinone is the major component of the essential oil but it is also present in the fixed oil [104]. The anticonvulsant activity of NS seed oil at a dose of 80 mg/kg was studied in the PIL model in rats. NS exerts anticonvulsant effects through multiple biochemical pathways and molecular interactions. The essential oil, particularly thymoquinone, modulates oxidative stress markers by increasing nitric oxide levels while decreasing glutathione (GSH) levels and catalase activity, indicating an oxidative stress response. Additionally, the NS seed oil decreases Na+, K + ATPase activity, which disrupts ion homeostasis and neuronal excitability, and increases acetylcholinesterase (AchE) activity, leading to altered cholinergic transmission. These combined effects contribute to the modulation of neural excitability and the anticonvulsant properties observed in the pilocarpine-induced seizure model [105].

### 5.10. *Martynia annua* (MA) Linn

MA (Martyniaceae) is a small herb native to Mexico, Central America, Burma, and West Pakistan and is found throughout India and is commonly known as the Cat's claw or Devil's claw. Phytochemical screening of the entire plant extract of MA exhibited the presence of carbohydrates, glycosides, phenols, tannins, flavonoids, and anthocyanins. Methanol leaf extract of MA showed the presence of a higher quantity of glycosides, alkaloids, terpenoids, and tannin steroids [106]. The anticonvulsant activity of MA is likely mediated by its enhancement of GABAergic neurotransmission. The extract's constituents, such as glycosides, alkaloids, terpenoids, and tannin steroids, potentially modulate GABA receptors or inhibit GABA degradation enzymes, leading to increased GABAergic activity. This augmentation of inhibitory signaling reduces neuronal excitability, resulting in a significant reduction in tonic hindleg extension in the MES model and a decreased duration of convulsions with delayed onset in the PTZ model [107].

### 5.11. *Trachyspermum ammi* (TA) Linn

TA (Apiaceae) is an annual herb and its small brown fruit is commonly known as Ajwain in Hindi. Thymol is the main component present in TA seeds. It also contains carvacrol, *p*-cymene, and *γ*-terpinene [108]. Methanol seed extract of TA was investigated at a dose of 50 mg/kg in STR seizures in rats. A significant decrease in the time of onset of convulsions and a decrease in duration of convulsions were noticed [109]. The anticonvulsant activity of TA is attributed to its constituent thymol, which enhances GABAergic neurotransmission. Thymol's ability to stimulate GABA responses, particularly by activating GABAA receptors and increasing chloride channel opening, leads to greater inhibitory signaling in the brain. This increase in GABA-mediated inhibition reduces neuronal excitability, resulting in a significant decrease in the time of onset and duration of convulsions in the STR seizure model. By modulating GABAergic pathways, thymol exerts its anticonvulsant effects, offering potential therapeutic benefits for the management of seizures and related neurological disorders [110].

### 5.12. *Ficus platyphylla* (FP)

FP (Meliaceae) commonly called peepal is mainly found in the savanna regions of the West African coast. Decoction of FP has been traditionally used in Nigeria to treat epilepsy, depression, psychosis, pain, and inflammation for many years. The anticonvulsant activity of methanol stem bark extract of FP at 100 and 200 mg/kg doses was studied in PTZ-induced seizures in albino mice. Extract improved seizure severity, cognitive deficits, and neuronal cell loss in this model. The anticonvulsant activity of FP methanol stem bark extract likely involves modulation of both GABAergic and glutamatergic neurotransmission pathways. The extract's components interact with GABA receptors, enhancing inhibitory neurotransmission, which reduces neuronal excitability and seizure severity. Additionally, FP extract may target undifferentiated glutamatergic receptors, mitigating excitatory neurotransmission and further stabilizing neural activity. By modulating both inhibitory and excitatory neurotransmitter systems, FP extract exerts its anticonvulsant effects, offering promise for the management of seizures and related neurological conditions [77].

### 5.13. *Bunium persicum* (BP)

*B. Fedtsch.* BP (Apiaceae) is a perennial aromatic plant, called wild caraway and is widely distributed in Pakistan, Tajikistan, Afghanistan, North India, and China. The high content of mono-terpenes and sesquiterpenes is found in the essential oil and extracts of BP [111]. Essential oil (0.25, 0.5, 1, 1.25, and 1.5 mL/kg) and methanol seed extract (3 and 4 g/kg) were evaluated against PTZ and MES-induced convulsions. BP has an acceptable safety profile as evidenced by acute toxicity and acute neurotoxicity study as no mortality was noticed at doses of 4 g/kg of extract and 2.5 mL/kg of oil. Essential oil significantly prevented tonic seizures in both models at doses of 1 mL/kg and higher doses. The methanol extract was effective against the PTZ convulsion model at a dose of 3 g/kg and no effect was found in MES-induced convulsion. Essential oil might be helpful to control absence and grand mal seizures at a dose of 1 mL/kg. BP exerts its anticonvulsant effects, particularly demonstrated in PTZ-induced convulsions, likely through modulation of GABAergic neurotransmission. The essential oil and methanol seed extract of BP interacts with GABAergic receptors, enhancing inhibitory neurotransmission, which reduces neuronal excitability and prevents the onset of seizures. This interaction with GABA receptors, especially noted with the essential oil, helps mitigate tonic seizures observed in both PTZ and MES-induced convulsions, suggesting potential efficacy in controlling various seizure types, including absence and grand mal seizures. By enhancing GABAergic transmission, BP offers promise as a natural therapeutic agent for managing epilepsy and related neurological disorders [112].

### 5.14. *Zingiber officinale (ZO)*

ZO (Zingiberaceae) popularly known as ginger is native to south-east Asia and cultivated in India, China, Nigeria, Indonesia, Bangladesh, and Thailand. The phytochemical study revealed that it contains terpenoids, tannins, essential oils, phenolic compounds, flavonoids, carbohydrates, proteins, alkaloids, glycosides, saponins, and steroids in major quantities [113]. The anticonvulsant effect of hydroethanolic rhizome extract at 25, 50, and 100 mg/kg doses was investigated in PTZ-induced seizure models in mice. A significant increase in the onset time of myoclonic seizure reduced generalized clonic and decreased forelimb tonic extension was reported. The anticonvulsant effect of ZO hydroethanolic rhizome extract in PTZ-induced seizure models likely involves modulation of multiple biochemical pathways and molecular interactions. ZO extract may interact with various types of calcium channels, regulating calcium influx and neuronal excitability, thus contributing to the observed increase in the onset time of myoclonic seizures and reduction in generalized clonic convulsions. Additionally, the extract may influence inhibitory and excitatory neurotransmitter systems, such as GABAergic and glutamatergic transmission, to stabilize neural activity and prevent seizure propagation. Moreover, ZO's antioxidant properties likely play a role in inhibiting oxidative stress, thereby protecting neurons from damage associated with seizures. By targeting these pathways and mechanisms, ZO extract offers the potential as a natural therapeutic agent for managing epilepsy and related neurological disorders [33].

### 5.15. *Viola betonicifolia (VB)*

VB (Violaceace) is a perennial herb or shrub, naturally found in India, Nepal, Sri Lanka, China, Malaysia, Pakistan, and Australia.The methanol extract of VB contains a high number of alkaloids, flavonoids, tannins, proteins, phenolic compounds, saponins, sterols, and triterpenoids. N-Hexane fraction of VB was evaluated for anticonvulsant activity at doses of 300, 400, and 500 mg/kg i.p. against PTZ and strychnine-induced seizure model in mice. Fraction protected the animals in a dose-dependent manner in PTZ but did not protect animals from convulsion in strychnine strychnine-induced seizure model. The anticonvulsant effect of VB n-hexane fraction against PTZ-induced seizures likely involves the excitation of GABA receptors. GABA receptors are pivotal in regulating neuronal excitability, and their activation leads to inhibitory neurotransmission, dampening excessive neuronal firing and preventing seizures. The n-hexane fraction of VB may contain compounds that interact with GABA receptors, either directly or indirectly, enhancing GABAergic neurotransmission and exerting anticonvulsant effects. However, the lack of protection against strychnine-induced seizures suggests that the mechanism of action may be specific to GABAergic pathways rather than affecting other neurotransmitter systems involved in strychnine-induced convulsions. This highlights the potential of VB as a source of GABAergic modulators for the management of epilepsy and related seizure disorders [30].

## 6. Ethnopharmacological Studies of Medicinal Plants With Antiepileptic Action

**Figure 2:**
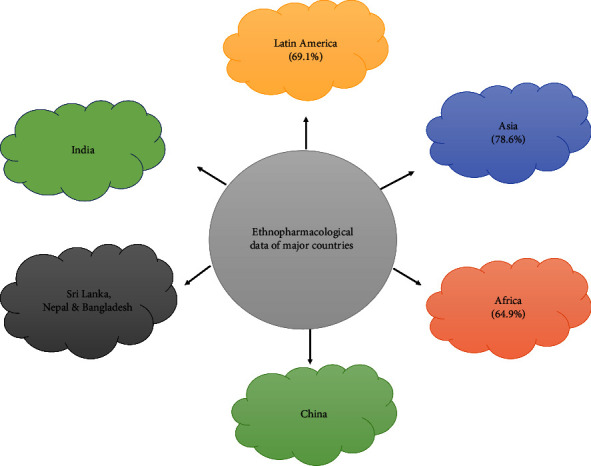
Major countries having traditional claims for antiepileptic action.

Despite the availability of well-established alternatives, traditional medicinal practices have remained a part of many civilizations' healthcare systems. However, the attention has changed to the use of herbal treatments in the therapy of epileptic seizures, possibly because these approaches fit with people's cultures, are less expensive, and have fewer side effects, contraindications, and probable combinations with other drugs. Herbal medicines and their active principles can be used as alternative medicine therapy for the treatment of this disease. The current study looked at certain medicinal plants from all over the globe that have been claimed to have antiepileptic and neuroprotective properties in the past (Table 2; Figure 2). Historical evidence suggests that herbal therapies have been used since 6000 BC in India for the treatment of epilepsy. Countries like China, Peru, and South America also have rich traditions in herbal therapies, including for convulsions [158, 159]. Various herbal and dietary therapies are also recommended for internal use, external application, and topical use in the nose and eyes, including Brahmirasayan, Ashwagandha, Brahmighritham, and old pure desi ghee, daily fresh juice of Brahmi with honey, powdered root of *Asparagus racemosus* with milk. Some other plants useful in epilepsy are *Withania somnifera, Bacopa monnieri, Acacia nilotica, Acorus calamus, Clitoreaternatea, Convolvulus pluricaulis, Emblica officinalis, Celastruspaniculatus,* and Muktapishti [160]. Herbal therapy is the used approach of complementary and alternative medications, which play an important role in the treatment of epileptic seizures [161].

## 7. Conclusion and Future Perspective

Research and development of herbal treatments for epilepsy have gained traction due to a growing preference for natural therapies. Preserving this ancient knowledge is crucial for discovering potential antiepileptic agents. This review aims to compile traditionally used plants for epilepsy treatment, along with the latest data on their active constituents. The information provided here, not previously available in this form, highlights 114 plant species as potential agents for epilepsy treatment. Traditional uses of these plants were verified from reliable sources such as books, articles, and reviews. Ten families stand out for their antiepileptic plants, with leaves often exhibiting the highest activity. Table 1 summarizes the most effective models for epilepsy. This data can guide researchers by providing insights into toxicity profiles, models, and study outcomes, potentially sparking further investigations into natural products for new antiepileptic agents. While many of the plants listed in Table 1 are yet to be experimentally studied on laboratory animals, those in Table 2 have shown potential in animal studies. However, their exact mechanisms of action and efficacy in humans remain largely unexplored, indicating a significant scope for further research on these lesser-known plants' chemical and biological activities for epilepsy treatment.

## Figures and Tables

**Table 1:** List of medicinal plants investigated for antiepileptic activity.

Botanical name (family)	Common name	Part used	Extract/compound/Dose	Models used	Results (efficacy)	Toxicity study	References
*Acorus calamus* Linn. (Araceae)	Vacha	Rh	Ethanol (250, 500 mg/kg, p.o.)	MES, PTZ	↓Hind limb extension and tonic flexion of forelimbs in MES. But failed to prevent mortality in PTZ suggesting inefficacy in petit mal epilepsy	No mortality at 750 mg/kg but clinical signs such as tremors, piloerection, and abdominal breathing were observed immediately after the oral dosing of 1750 mg/kg	[24, 25]
*Achyranthes aspera* Linn. (Amaranthaceae)	Chaff –flower, Devil's horsewhip	R	Methanol (2.5, 5, and 10 mg/kg, i.p.)	PTZ, MES, and PIC	Dose-dependent anticonvulsant effect in PTZ and PIC but did not show the protection in MES	LD_50_ 200 mg/kg	[26, 27]
*Vetiveria zizanioides* Linn. (Gramineae)	Vetiver	R	Ethanol (100, 200, and 400 mg/kg)	MES and PTZ	Hind limb extension in the MES model only	LD_50_ 600 mg/kg	[28]
*Viola betonicifolia* (Violaceae)	Arrowhead violet, mountain violet	Wp	Methanol (300, 400, and 500 mg/kg)	MES and STR in rats	Significant results at 400 and 500 mg/kg in the PTZ model, all animals were found dead in the STR model	Safe up to 2000 mg/kg	[29, 30]
*Ficus religiosa* (Moraceae)	Bodhi tree	Fg	Methanol (25, 50, and 100 mg/kg i.p.)	MES, PIC, and PTZ	↑ threshold of MES and PIC-induced convulsions with no neurotoxic effects	Safe up to 2000 mg/kg	[31, 32]
*Zingiber officinale* (Zingiberaceae)	Ginger	Rh	Hydroethanol (25, 50, and 100 mg/kg i.p.)	PTZ	↑ onset time of myoclonic seizure, prevent generalized clonic, and ↑ threshold for forelimb tonic extension	LD_50_ > 1500 mg/kg	[33, 34]
*Punica granatum* Linn. (Punicaceae)	Pomegranate	S	Ethanol (150, 300, and 600 mg/kg p.o.)	PTZ and STR	↑seizure latency at two higher doses in both models	LD_50_ > 2000 mg/kg	[35, 36]
*Calotropis procera* (Ait.) R.Br (Asclepiadaceae)	Auricula tree	R	Pet-ether, alcohol, aqueous (500 mg/kg and chloroform 200 mg/kg i.p.)	MES, PTZ, L-PIL, and electrically kindled in rats	In the MES model chloroform extracts ↓the duration of hind limb extension; and delayed the onset of convulsion in PTZ; chloroform extracts ↑ the latency of seizure in L-PIL and electrically kindled model.	LD_50_ > 3000 mg/kg	[37, 38]
*Hibiscus rosasinesis* (Malvaceae)		F	Pet-ether, chloroform, alcohol, and aqueous (250 mg/kg, i.p.)	MES and ISO	Alcohol extracts↓ the duration of the hind limb, more efficacious than another extract the in MES model. However, in the ISO model 100% mortality was observed.	LD_50_ 1600 mg/kg	[39, 40]
*Calotropis gigantea* Linn. (Asclepiadaceae)	Crown flower	L	Pet-ether, benzene, chloroform (200 mg/kg), methanol (180 mg/kg), and aqueous (200 mg/kg)	MES, PTZ	Methanol extract revealed anticonvulsant activity in both MES and PTZ models than other extracts	LD_50_ 3 gm/kg	[41, 42]
*Boerhaavia diffusa* Linn. (Nyctaginaceae)	Red hogweed, Punarnava	R	Methanol (1000, 1500, and 2000 mg/kg, i.p.)	PTZ	Protection was observed	LD_50_ > 2000 mg/kg	[43, 44]
*Cissus quadrangularis* (Vitaceae)	Hadjod, Veldt grape	Wp	Aqueous (37.22, 93.05, 186.11, and 372.21 mg/kg, p.o.)	PIL	↑ latency of clonic, generalized tonic-clonic seizures; ↓the number and duration of seizures in a dose-dependent manner	Safe up to 3000 mg/kg	[45, 46]
*Cardiospermum halicacabum* (Sapindaceae)	Kanphuti	R	Alcohol (30, 100, and 300 mg/kg, p.o.)	MES, PTZ, PIC, STR, and ISO	MES showed antiepileptic effect; ↑ Duration of onset of tonic-clonic convulsions in PTZ model; PIC at higher dose produced ↑ duration of onset of tonic-clonic convulsions; 100 mg/kg produced prolongation of onset of tonic convulsion in STR; ISO at dose 300 mg/kg delayed the onset of clonic and tonic convulsion.	Safe up to 2000 mg/kg	[47, 48]
*Cocos nucifera* Linn. (Arecaceae)	Coconut	R	Aqueous (50 and100 mg/kg, p.o.)	MES	Significantly delayed the onset of tonic convulsions and ↓the duration of seizures at both doses.	Safe up to 2000 mg/kg	[49, 50]
*Psidium guajava* (Myrtaceae)	Guava	L	Ethanol (200 and 400 mg/kg)	MES and PTZ	400 mg/kg ↓ duration of tonic hind limb extension	LD_50_ > 5000 mg/kg	[51, 52]
*Taxus wallichiana* Zucc. (Taxaceae)	Himalayan Yew	L	Methanol (50, 100, and 200 mg/kg, i.p., s.c)	PTZ	The extract in all three doses through both routes fully protected the mice from tonic-clonic seizures.	LD_50_ > 2000 mg/kg	[53]
*Cynodon dactylon* (Poaceae)	Bermuda grass, Dhoob	Wp with R	Ethanol (200, 400, and 600 mg/kg oral)	PTZ in mice and MES in rats	400 and 600 mg/kg ↓ duration of convulsion	LD_50_ > 2000 mg/kg	[54, 55]
*Adhatoda vasica* (Acanthaceae)	Malabur nut, Vasaka	L	Aqueous (100 and 200 mg/kg, i.p.)	MES	Dose dependant protection from seizures	LD_50_ > 3200 mg/kg	[56, 57]
*Aegle marmelos* (Rutaceae)	Bael	L	Ethanol (50, 100, and 200 mg/kg, i.p.)	PTZ, MES	↓ severity and duration of seizure	Safe up to 6 gm/kg	[58, 59]
*Albizia lebbeck* Linn. (Fabaceae)	Siris tree, woman's tongue	L	Ethanol (200 and 400 mg/kg, p.o.)	MES and PTZ	Delayed onset of convulsions in PTZ model while reduction in tonic hind limb extension in MES model	LD_50_ 2,000 mg/kg	[60, 61]
*Allium cepa* Linn. (Liliaceae)	Onion	Bu	Methanol (8 mg/20 g and 16 mg/20 gs (p.o.)	MES	Delayed hind limb tonic extension and	Safe up to 3,000 mg/kg	[62, 63]
*Anacyclus pyrethrum* (Asteraceae)	Spanish chamomile	T	Hydroalcohol (50, 100, 250, 500, and 1000 mg/kg, oral)	MES, PTZ	Latency of myoclonic jerks significantly ↑ in PTZ.	Safe upto 5000 mg/kg	[64, 65]
*Apium graveolens* Linn. (Umbelliferae)	Ajmod	Ap	Aqueous extract (100, 500, and 1000 mg/kg, i.p.)	PTZ in rats	↑ Minimal clonic Seizure and generalized tonic-clonic Seizure latencies	Safe up to 2000 mg/kg	[66, 67]
*Artemisia indica* Linn. (Asteraceae)	Mugwort	Wp	Methanol extract (10, 30, and 100 mg/kg, i.p.)	PTZ in mice	↑ the duration of onset of tonic-clonic convulsions and ↓ duration of convulsions	Safe up to 200 mg/kg	[68]
*Artemisia nilagirica* (Asteraceae)	Indian worm wood	L	Ethanol, diethyl ether and chloroform extracts/100, 200, 400, 600, 800, and 1000 mg/kg, oral)	PTZ in mice	Among the extracts ethanolic extract (600 and 800 mg/kg) produced better anticonvulsant activity than chloroform extract. Diethyl ether extract did not produce anticonvulsant activity.	Safe up to 2 gm/kg	[69, 70]
*Azima tetracantha* (Salvadoraceae)	Bee sting bush, needle bush	R	Ethanolic extract (250 and 500 mg/kg, oral)	MES and PTZ in mice	500 mg/kg of extract has better protection than 250 mg/kg against seizure in the MES model and is equally efficacious as sodium valproate in the PTZ model	Safe up to 2000 mg/kg	[71]
*Commiphora wightii* (Burseraceae)	Guggul	B	Oleo gum resin extract (200 and 400 mg/kg, oral)	PTZ in mice	Extract at both doses delayed the onset of convulsion	Safe up to 3 gm/kg	[72, 73]
*Coriandrum sativum* (Apiaceae)	Coriander	Ap	Hydroalcoholic extract (100, 500, and 1000 mg/kg, oral)	PTZ in rats	Extract showed protection against seizures and oxidative stress induced by PTZ model	More than 2000 mg/kg	[74, 75]
*Terminalia chebula* (Combretaceae)	Myrobalan	F	Ethanol extract (200 and 500 mg/kg, p.o.)	MES, PTZ, and PIC in mice	Significantly ↓ the duration of the seizures in MES model In PTZ model significantly delayed the latency of the seizures Extract at both doses did not affect the incidence of seizures, but significantly prolonged the latency of seizures in the PIC model	Safe up to 2000 mg/kg	[76]
*Ficus platyphylla* (Moraceae)	Broad leaf fig	St,B	Methanol extract (100, 200, and 400 mg/kg, i.p.)	PTZ, STR, PIC, and ISO in mice	Extract showed significant result at a dose of 400 mg/kg in STR model	LD_50_ above 3000 mg/kg	[77, 78]
*Gossypium herbaceum* (Malvaceae)	Levant cotton	L	Aqueous extract (10, 30 and 100 mg/kg, p.o.)	MES, PTZ, and ISO in mice	Extract exhibited significant dose-dependent antiepileptic activity in both MES and PTZ induced convulsion model	LD_50_ 2000 mg/kg	[79]
*Desmodium triflorum* Linn. (Fabaceae)	Black clover	L	Ethanolic extract (400 and 800 mg/kg p.o.)	PTZ, ISO, and MES in mice	Extract at the doses of 400 and 800 mg/kg showed significant delayed onset of convulsion against PTZ, ISO, and MES model	Safe up to 2000 mg/kg	[80]
*Cyperus esculentus* Linn. and *Cyperus rotundus* Linn (Cyperaceae)	Nut grass	Eo	(250 and 500 mg/kg, p.o.)	MES in rats	Extract with higher dose exhibited a significant anticonvulsant activity	Safe up to 5000 mg/kg	[81]
*Nardostachys jatamansi* (Valerianaceae)	Jatamansi	R	Ethanolic extract (100, 200, and 400 mg/kg, i.p.)	PTZ, ISO, and MES in rats	Extract at higher doses decreased tonic hind limb extension in the MES model and exhibited significant prolongation of seizure latency at doses of 200 and 400 mg/kg in the PTZ model.	Safe up to 2000 mg/kg	[82, 83]
*Nigella sativa* Linn. (Ranunculaceae)	Black cumin	S	Oil (80 mg/kg, i.p.)	PTZ in mice	Exhibited significant anticonvulsant activity	(LD50 value 28.8 L/kg body wt., p.o.; LD50 value 2.06 mL/kg body wt., i.p.)	[84]
*Oroxylum indicum* (Bignoniaceae)	Broken bones plant	L	Methanolic extract (200 mg/kg, i.p.)	MES and PTZ in rats	In PTZ model extract showed a significant reduction in myoclonic jerk and tonic flexation Extract showed a significant decrease in tonic convulsion and clonic in the MES model	Lethal dose more than 2000 mg/kg	[85, 86]
*Picrorhiza kurroa* (Scrophulariaceae)	Kutki	R	Ethanolic extract (25, 50, and 100 mg/kg)	PTZ, MES, and PIC in mice	Extract at higher dose increased the latency of clonic convulsions in PTZ while in MES reduced 100 mg/kg significantly increased latency to clonic convulsions and reduced incidence of tonic hind limb extension without significant effect on mortality	More than 2000 mg/kg	[87, 88]
*Piper longum* (Piperaceae)	Long pepper	F	Piperine (30, 50, and 70 mg/kg, i.p.)	PTZ and PIC in mice	Piperine protected animals from PTZ-induced seizures in a dose-dependent manner, significantly increased the PIC-induced latency of convulsion	Safe up to 5000 mg/kg	[89, 90]
*Ricinus communis* Linn. (Euphorbiaceae)	Castor	L	Ethanolic extract (200 and 400 mg/kg, Oral)	MES and PTZ in rats	Extract at both doses showed a significant reduction in the duration of convulsion in a dose-dependent manner	Greater than 2000 mg/kg	[91, 92]

Abbreviations: Ap, aerial part; B, bark; Bu, bulb; Eo, essential oil; F, fruit; Fg, fig; Fl, flower; ISO, isoniazid; L, leaves; LD, lethal dose; MES, maximal electroshock seizure; PIC, picotoxin; PIL, pilocarpine; PTZ, pentylenetetrazole; R, root; Rh, rhizome; S, seed; St, stem; STR, strychnine; Wp, whole plant.

**Table 2:** List of traditional medicinal plants used for the treatment of Epilepsy.

Botanical name	Family	Common name	Geographical source	Part used	Tribal use	References
*Achillea millefolium*	Asteraceae	Worm Wood	Asia, middle east, Europe	H	ND	[114]
*Acorus calamus* Linn.	Acoraceae	Sweet flag	India (Manipur Kashmir, Nagaland)	Rh R	Washed fresh rhizome paste prescribed twice a day orally	[115]
*Allium sativum* Linn.	Liliacceae	Garlic	Egypt, Greece, China, and India	Bu	Bulb is boiled in water till the extract is reduced to half of its volume; a half cup of the filtered extract is given internally.	[116]
*Adhatoda vasica*	Acanthaceae	Vasaka	India, Srilanka, Burma, and Malaysia	L, R, Fl, B	ND	[56]
*Adiantum lunulatum* Linn.	Pteridaceae	Walking maiden hair fern	India	R	ND	[117]
*Allium cepa* Linn.	Liliaceae	Onion	India	Bu, S	Extract of onion bulb and mentha leaves is given orally for a week	[116]
*Alsotonia venenatus* R.Br	Apocynaceae	Devil tree	Forests of India	F, R	ND	[118]
*Anacyclus pyrethrum*	Asteraceae	Pellitory and Akarkara	India, North Africa, Arabia, Syria,	R	ND	[119]
*Anagallis arvensis* Linn.	Primulaceae	Biliputi	Europe, Asia, US, nontropical south America	H	ND	[120]
*Aplotaxis auriculata*	Compositae	Saussureaauriculata		L	Half a tola of the fresh juice of leaves boiled with ghee and formed into a ghrita or mixed with two scruples of roots of plant and honey is given in epilepsy	[118]
*Artemesia absinthium* Linn.	Asteraceae	Wormwood	Asia, middle east, North Africa and Europe	H, L	ND	[121]
*Artemisia indica* Linn.	Asteraceae	Mugwort	Europe, Asia, northern Africa	Wp	ND	[68]
*Asparagus racemous* Willd	Liliaceae or Asparagaceae	Shatavari	Australia, Sri Lanka, China, India	R, L	Roots with honey and cow's milk	[122]
*Benincasa cerifera*	Cucurbitaceae	White guard, ash pumpkin	India, south-east Asia, and east Asia	S, Fr, FrJ	Fruit with cow's milk used to treat epilepsy	[123]
*Brassica alba*	Cruciferae	Sufedrai	India	S, O	ND	[124]
*Camphora officinarum*	Lauraceae	Duk	India	Vo	ND	[124]
*Canscora decussata* Roem and Schult.	Gentianaceae	Shankhpushpi	India	Wp, J	ND	[125]
*Vitis quadrangularis*	Vitaceae	Hadjora, Harjora	India, east Africa	St	ND	[118]
*Cardamine pratensis* Linn.	Brassicaceae	Cuckooflower	Europe, north America, India, west Asia	Fl	ND	[118]
*Careya arborea* Roxb	Lecythidaceae	Wild guava	India, Ceylon, Malay, and Peninsula	B	ND	[126]
*Cassia occidentalis* Linn.	Caesalpiniaceae	Kasaundi	India	S	ND	[69]
*Cedrus deodara* Roxb	Pinaceae	Deodar	Northern Pakistan, north-central India, southwestern Tibet, and western Nepal	L	ND	[127]
*Centratherum anthelminticum* Linn.	Asteraceae	Black cumin or Kalijiri	India, southest Asia	Wp	ND	[118]
*Citrus maxima* Linn.	Rutaceae	Chakotra, Pummelo	India, China, Asia, Thailand	L, F	ND	[128]
*Clerodendrom serratum* Linn.	Verebenaceae	Bharangi	India	R	ND	[129]
*Cocculus suberosus*	Menispermaceae	Duk	India	FFr	ND	[124]
*Colebrookea oppositifolia* Sm.	Lamiaceae	Seniya or sanya Binda	India, China Myanmar, Nepal,	R	Half cup decoction of roots is prescribed once a day for 2 weeks or more	[118]
*Coleus ambaoinicus*	Lamiaceae	Ajavayin, Patharcur	India	D, Ej	ND	[118]
*Curcuma zedoaria*	Scitaminaceae	Kalihaladi	India	Rh	ND	[118]
*Cymbopogon citrates* (DC) Stapf	Poaceae	Citronella grass or lemongrass	India, Sri Lanka and south-east Asia	Gr	Leaf paste is taken to treat epilepsy	[130, 131]
*Cymbopogon schoenanthus*	Poaceae	Camel grass	Eastern Africa, Iran	Gr	ND	[132]
*Cynodon dactylon* Linn.	Poaceae	Doorva	India, Africa	Wp, Ej	ND	[133]
*Cyperus rotundu* Linn.	Cyperaceae	Motha, Mutha	Africa, Europe, and Asia	R	ND	[134]
*Cyperuss cariosus*	Cyperaceae	Nagarmotha	Bangladesh	R	ND	[135]
*Cypraea moneta* Linn.	Cypraeidae	Kaudi	India	—	ND	[124]
Datura alba	Solanaceae	Dhattura	India	Wp	ND	[136]
*Datura fastuosa*	Solanaceae	Thorn Apple,	India	S, L	ND	[136]
*Elaceocarpus genitus*	Elacocarpaceae	Rudraksha	India, Nepal, Indonesia, Java,	F	Pulverized bark and pulp of fruit or the bead can be used to cure epilepsy	[137]
*Elaeocarpu stuberculatus* Roxb.	Elaeocarpaceae	Rudrak	Peninsula Malaya	N	ND	[118]
*Eleusine indica* Linn.	Poaceae	Malankuri	India, Ceylon	D	ND	[118]
*Ervatamia coronaria* Stapf	Apocynaceae	Crepe jasmine	India	O	ND	[138]
*Evolvulus alsinoides* Linn.	Convolvulaceae	Shankhpuspi	India	H	About 10 g of plant paste is mixed with 100 g of curd made by cow's milk, given orally once a day for 1 month to treat epilepsy	[139]
*Excoecaria agallocha*	Euphorbiaceae	Blinding Tree	Tropical Africa, Asia and Australia	L	ND	[118]
*Flemingia chappar*	Fabaceae	Salpan	China, India	R	ND	[140]
*Flemingia strobilifera* R.Br.	Fabaceae	Luck plant kusrunt,	Burma, India	R	ND	[140]
*Humboldtia vahliana*	Caesalpiniaceae	Malabar Humboldtia	India	B	ND	[141]
*Ipomea hispida* Roem. and schult.	Convolvulaceae	Small rabbit root	India	O	ND	[118]
*Limonia crenulata*	Rutaceae	Beli	India	L	ND	[118]
*Pinus longifolia* Roxb.	Pinaceae	Chir pine	Bhutan, India	G	ND	[118]
*Martynia annua* Linn.	Martyniaceae	Cat's claw	India, Burma, West Pakistan	L	Half cup decoction of leaves given once a day	[142]
*Matricaria chamomilla* Linn.	Asteraceae	German chamomile	Japan, N. Asia	Fl	ND	[118]
*Mimosa pudica*	Fabaceae	Touch-me-not plant	India, tropical America	Wp	Macerated root is prescribed thrice a day	[131]
*Moniera cuneifolia*	Plantaginaceae	Brahmi	India, Ceylon	—	ND	[118]
*Musa sapeintum*	Musaceae	Banana tree	India, China, Mexico	L,UFr	ND	[143]
*Valeriana officinalis* Linn.	Valerianaceae	Common valerian	Europe, north and west Asia	R	ND	[118]
*Myrtus communis*	Myrtaceae	Murad	India	L	ND	[118]
*Nardostachys jatamansi* DC	Valerianaceae	Balchara, jatamansi	India and Bhutan	R	ND	[144]
*Paeonia emodi* Wall. ex Royle	Paeoniaceae	Himalayan Peony	India (western Himalayan region)	Tu	Root powder is mixed with Selinum vaginatum root powder and given 1/2 teaspoon twice a day up to 6 months	[145]
*Picrorrhiza kurrooa* Royle ex Benth	Scrophulariaceae	Katki, Kuru	India	R (white)	ND	[118]
Saussurea lappa	Compositae	Kuth root	India	R	ND	[118]
*Sapindus trifoliate* Linn.	Sapindaceae	Ritha	India	R	ND	[118]
*Solanum nigrum* Linn.	Solanaceae	Makoi	Africa, Indonesia	Wp	ND	[146]
*Sphaeranthus indicus* Linn.	Asteraceae	Mundi	India	H	ND	[147]
*Strychnos bourdilloni*	Loganiaceae	—	China, India, SriLanka, Bangladesh, Vietnam, Indonesia	D of R	ND	[118]
*Strychnos cinnamomifolia*	Loganiaceae	—	India	D of R	ND	[118]
*Strychnos nuxvomica*	Loganiaceae	Kupila or Kuchila	India	F, B	ND	[148]
*Taxus baccata*	Taxacea	Common yew, European yew	North Africa, Turkey	L	ND	[118]
*Trichosanthes palmata* Roxb.	Cucurbitaceae	Mahakal, Lalindravam	Japan, China	F	ND	[118]
*Trema orientalis* Linn.	Ulmaceae	Pigeon wood, Charcoal tree	India, Bangladesh, Nepal	L	ND	[149]
*Valeriana wallichii*	Valerianaceae	Tagar	India, Bhutan	R	ND	[150]
*Verbena officinalis* Linn.	Verbenaceae	“Vervian,” “herb of grace,” “Pigeon's grass	Europe and Asia	Ap	ND	[151]
*Rauwolfia serpentina* Benth	Apocynaceae	Sarpgandha	India	R	ND	[152]
*Xanthium strumarium*	Compositae	Cocklebur	North America, Brazil, China, Malaysia and India	Wp	ND	[153]
*Ricinus communis* Linn.	Euphorbiaceae	Castor	Africa and India	L	The 1–2 teaspoonful infusion of leaves is administered internally, once, or twice a day.	[116]
*Bacopa monnieri* Linn.	Scrophulariaceae	Brahmi	India and neighboring tropical countries	H	Half cup decoction of leaves pastes or juice is used internally, taken twice or thrice a day	[116]
*Achyranthes aspera*	Amaranthaceae	Chaff–flower, Devil's horsewhip	Ceylon, America, Australia	Ap	The whole plant is mixed with 6–7 grains of dried grapes and crushed to about 15–20 g of powder. One teaspoon of powder prescribed orally twice a day	[139]
*Dryopteris cochleate*	Dryopteridaceae	Wood ferns, male ferns	Asia, Europe, Africa	Rh	The rhizome of the plant is crushed into powderandgivenwith water twice a day	[154]
*Cissus quadrangularis* Linn.	Vitaceae	Veldt grape, devil's backbone, hadjod	Tropical Asia, India, Arabica	St	Stem crushed in water, taken orally twice a day for 5 days	[122]
*Punica granatum* Linn.	Lythraceae	Anar	India, central, and southeast Asia	L	Leaves boiled with half liter of water and 10 rose leaves; the extract is reduced to half of its volume. Filtered extract with some butter is prescribed for the treatment of epilepsy	[155]
*Acacia farnesiana* Linn. Wild	Fabaceae	Cassie flower, cassie	South America, India, Nepal Pakistan, Sri Lanka	B, L, Hw G, R	ND	[118]
*Catharanthus pusillus* (Murr.) G. Don	Apocynaceae	Tiny Periwinkle, Tiny Vinca	India	Wp	ND	[118]
*Boerhavia diffusa* Linn.	Nyctaginaceae	Punarnava	Nigeria, India, tropical Africa	Ap	Decoction of aerial parts is prescribed	[156]
*Vitex negundo*	Verbenaceae	Chinese chaste tree	China, India, Japan	R, B	Root bark is ground and mixed with local liquor and form a paste, applied over neck	[157]

Abbreviations: Ap, aerial parts; B, bark; Bu, bulb; D, decoction; Ej, expressed juice; Eo, essential oil; F, fruit; FFr, fresh fruit; Fl, flower; Fg, fig; Frj, fruit juice; G, gum; Gr, grass; H, herb; Hw, heartwood; J, juice; L, leaves; N, nuts; ND, not documented; O, oil; R, root; Rh, rhizome; S, seed; St, stem; Tu, tubers; Ufr, unripe fruit; Wp, whole plant.

## Data Availability

Information/data collected from open source.

## Nomenclature

CDC: Centers for disease control and prevention
CNS: Central nervous system
GABA: Gamma-aminobutyric acid
GSH: Glutathione
GTCS: Generalized tonic-clonic seizure
MCS: Minimal clonic seizure
MES: Maximal electroshock seizure
NMDA: N-methyl-D-aspartate
PTZ: Pentylenetetrazole
WHO: World Health Organization
